# Bioinformatics analysis of the key potential ceRNA biomarkers in human thymic epithelial tumors

**DOI:** 10.1097/MD.0000000000026271

**Published:** 2021-06-18

**Authors:** Kegong Chen, Long Bai, Lin Ji, Libo Wu, Guanghua Li

**Affiliations:** a Department of Cardiovascular Surgery, The Second Affiliated Hospital of Harbin Medical University; b Key Laboratory of Myocardial Ischemia, Ministry of Education, Harbin Medical University; c Department of Orthopedics, The First Hospital of Harbin, Harbin Institute of Technology; d Department of Thoracic Surgery, The Second Affiliated Hospital of Harbin Medical University, Harbin, Heilongjiang; e Department of Chest Surgery, Affiliated Cancer Hospital and Institute of Guangzhou Medical University, Guangzhou, China.

**Keywords:** biomarkers, ceRNA network, prognostic, thymic epithelial tumors

## Abstract

**Background:**

Thymic epithelial tumors (TETs), originating from the thymic epithelial cells, are the most common primary neoplasms of the anterior mediastinum. Emerging evidence demonstrated that the competing endogenous RNAs (ceRNAs) exerted a crucial effect on tumor development. Hence, it is urgent to understand the regulatory mechanism of ceRNAs in TETs and its impact on tumor prognosis.

**Methods:**

TETs datasets were harvested from the UCSC Xena as the training cohort, followed by differentially expressed mRNAs (DEmRNAs), lncRNAs (DElncRNAs), and miRNAs (DEmiRNAs) at different pathologic type (A, AB, B, and TC) identified via DESeq2 package. clusterProfiler package was utilized to carry out gene ontology and Kyoto encyclopedia of genes and genomes functional analysis on the DEmRNAs. Subsequently, the lncRNA-miRNA-mRNA regulatory network was constructed to screen the key DEmRNAs. After the key DEmRNAs were verified in the external cohort from Gene Expression Omnibus database, their associated-ceRNAs modules were used to perform the K-M and Cox regression analysis to build a prognostic significance for TETs. Lastly, the feasibility of the prognostic significance was validated by receiver operating characteristic (ROC) curves and the area under the curve.

**Results:**

Finally, a total of 463 DEmRNAs, 87 DElncRNAs, and 20 DEmiRNAs were obtained from the intersection of differentially expressed genes in different pathological types of TETs. Functional enrichment analysis showed that the DEmRNAs were closely related to cell proliferation and tumor development. After lncRNA-miRNA-mRNA network construction and external cohort validation, a total of 4 DEmRNAs DOCK11, MCAM, MYO10, and WASF3 were identified and their associated-ceRNA modules were significantly associated with prognosis, which contained 3 lncRNAs (lncRNA LINC00665, lncRNA NR2F1-AS1, and lncRNA RP11-285A1.1), 4 mRNAs (DOCK11, MCAM, MYO10, and WASF3), and 4 miRNAs (hsa-mir-143, hsa-mir-141, hsa-mir-140, and hsa-mir-3199). Meanwhile, ROC curves verified the accuracy of prediction ability of the screened ceRNA modules for prognosis of TETs.

**Conclusion:**

Our study revealed that ceRNAs modules might exert a crucial role in the progression of TETs. The mRNA associated-ceRNA modules could effectively predict the prognosis of TETs, which might be the potential prognostic and therapeutic markers for TETs patients.

## 1 Introduction

Thymic epithelial tumors (thymoma and thymic carcinoma, TETs) were the most common primary tumors of anterior mediastinum in humans, which originated from thymic epithelial cells. In light of the 2015 World Health Organization (WHO) classification, TETs were categorized as thymomas (A, AB, B1, B2, and B3 subtypes) and thymic carcinomas (TCs) based on the tumor cell morphology, degree of atypia, and extent of the thymocyte component.^[1]^ Thymoma has the basic structure of the thymus, whereas the tissue structure in TC is unclear. The WHO histological classification of thymoma is closely related to its prognosis. Type A, AB, and B1 thymoma progress slowly, and the 5 to 10 years overall survival rate is more than 80%.^[2]^ While thymomas B2 and B3 have an intermediate behavior, and their 5-year survival rate is close to 50%.^[3]^ Early TETs can be treated surgically, but the advanced and recurrent TETs have only palliative therapy with chemotherapy. Although targeted therapy had shown promising results in other types of tumors, the advance of targeted therapy in TETs is still slow.^[4–6]^ Therefore, screening the biomarkers for early identification and potential molecular targets for advanced and recurrent TETs was greatly significant.

The ceRNAs competitively bind specific miRNAs with other RNA molecules to regulate the expression of target genes.^[7–9]^ Considering that any transcripts containing miRNA response elements can theoretically function as ceRNAs, they may represent a widespread form of post-transcriptional regulation of gene expression, whether in pathological or physiological situations. The activity of ceRNA could be affected by many factors, such as the abundance, subcellular localization, the binding affinity of miRNAs to sponge, RNA secondary structure, and RNA binding protein. The distortions of these factors may release the control of the ceRNA network, implicated in human diseases, including cancer.^[10]^ The endogenous RNA molecules with miRNA action sites can compete with miRNA combinations, thereby indirectly regulating the expression of miRNA target genes. This competitive binding miRNA role is called miRNA molecular sponges.^[11]^ It has been shown that various RNA molecules (including mRNA, lncRNA, miRNA, circular RNA, and Pseudogenes) could be mutually regulated through the ceRNAs mechanism, thereby affecting the proliferation and metastasis of the tumors.^[12]^ In addition, ceRNAs are also considered as a specific mechanism involved in the regulation of tumor-related proteins.^[13]^ Therefore, the research for ceRNAs molecular related to TETs would provide new perspectives on the diagnosis and treatment of TETs.

In this study, we comprehensively analyzed and identified the differentially expressed mRNA (DEmRNAs), lncRNAs (DElncRNAs), and miRNAs (DEmiRNAs) that were associated with TETs progression. The potential key DEmRNAs were screened via lncRNA-miRNA-mRNA network and their ceRNAs modules. The K-M and Cox regression analysis were used to assess the prognostic significance for TETs. The predictive accuracy was validated by receiver operating characteristic (ROC) curves and area under the curve (AUC) validation. The study identified the novel diagnostic ceRNAs modules of TETs and provided new evidence for the early screening and therapeutic target for TETs patients.

## 2 Materials and methods

### 2.1 Data collection

**Figure 1:**
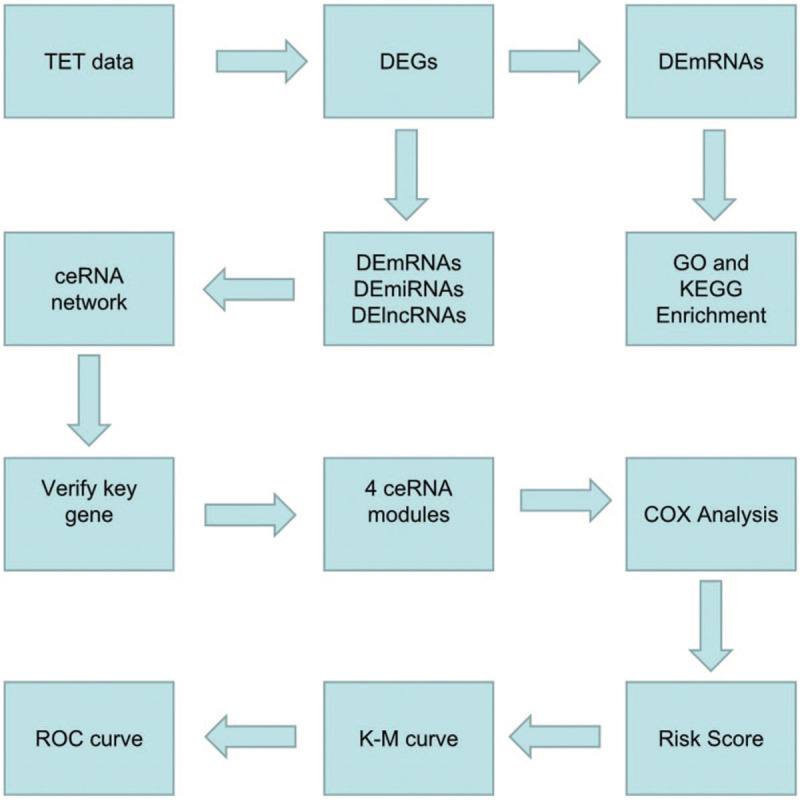
The flow chart of the present study.

The mRNAs, lncRNAs, and miRNAs expression profiles of TETs were downloaded from the UCSC Xena (https://xena.ucsc.edu/) database, containing 16 samples of type A, 35 samples of type AB, 57 samples of type B, 11 samples of TC, as well as 2 samples of normal tissue. The flow chart of the study was presented in Fig. 1. And our study had been approved by the ethics committee of The Second Affiliated Hospital of Harbin Medical University.

### 2.2 Differently expression genes identification

Firstly, the genes that were expressed in less than 10% of the samples would be eliminated. Next, the R package DESeq2^[14]^ was used to screen the differently expression genes (DEGs) between normal tissues and different pathologic type of TETs (A, AB, B, and TC), under the threshold of false discovery rate (FDR) < 0.25 and Log_2_ (Fold Change (FC)) > 1.

### 2.3 GO and KEGG pathway annotation of DEmRNAs

Gene ontology (GO) and Kyoto Encyclopedia of Genes and Genomes (KEGG) functional enrichment analysis could be used to investigate the potential biological function of the target multigene or geneset. In our study, the R package clusterProfiler (enrichGO and enrichKEGG function)^[15]^ was used to conduct the GO and KEGG enrichment analysis. *P* < .05 was considered to be significant.

### 2.4 Construction of ceRNAs network

The lncRNA-miRNA-mRNA interaction network was constructed via Cytoscape according to the relationship between lncRNA, mRNA, and miRNA. The criteria adopted to determine the ceRNA relationship was as follows: (1) mRNAs and lncRNAs were competitively bound to miRNA. The hypergeometric method used to verify the enrichment significance of miRNAs between mRNA and lncRNA via the following formula (*P* < .05)

$$p=\sum_{i=c}^{\min(K,n)}\frac{\left(\begin{array}{c} K\\ i \end{array}\right)\left(\begin{array}{c} N-K\\ n-i \end{array}\right)}{\begin{array}{c} N\\ n \end{array}}$$

(*N* = the total number of miRNAs, *K* = the number of miRNAs targeting mRNA, *n* = the number of miRNAs targeting lncRNA, and *c* = the number of miRNAs shared by the mRNA and lncRNA). (2) There was a positive correlation between the expression of mRNAs and lncRNAs (correlation ≥0.4 and *P*-value <.05).

### 2.5 Verification of DEmRNAs in an external validation cohort

To further verify the reliability of the results, an external cohort of GSE79978 from Gene Expression Omnibus (GEO) database which contained 13 samples of TETs and 3 samples of the normal thymus, was utilized to perform the DEGs analysis via the Wilcoxon Rank sum test. (*P* < .05).

### 2.6 Overall survival analysis and establishment of the TETs-specific prognostic significance

To explore the relationship of selected ceRNAs modules with the prognosis of TETs patients, the survival analysis was conducted via Kaplan–Meier survival analysis and log-rank test (cutoff point: median value). Univariate cox proportional hazards regression was used to identify the relationship between lncRNA-miRNA-mRNA and overall survival. For each ceRNA module, the multivariate survival analysis risk score was calculated to determine the impact of ceRNA on the prognosis of TETs via the survival package in R. In addition, the survival ROC package^[16]^ was utilized to build the ROC curves and estimate the area under the ROC curve (AUC) for the 4 ceRNA models to evaluate the accuracy for the prediction of prognosis.

## 3 Results

### 3.1 Differential RNA expression analysis in TETs

**Figure 2:**
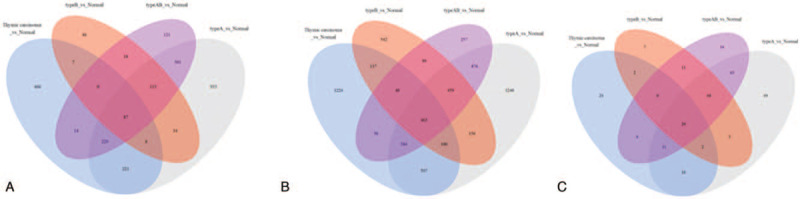
Significantly expressed genes in different pathologic type TETs. (A) The Venn diagram for DElncRNAs. (B) The Venn diagram for DEmRNAs. (C) The Venn diagram for DEmiRNAs (Log_2_FC > 1, FDR < 0.25). TETs = thymic epithelial tumors, DElncRNAs = differentially expressed lncRNAs, DEmRNAs = differentially expressed mRNA, DEmiRNAs = differentially expressed miRNA.

**Figure 3:**
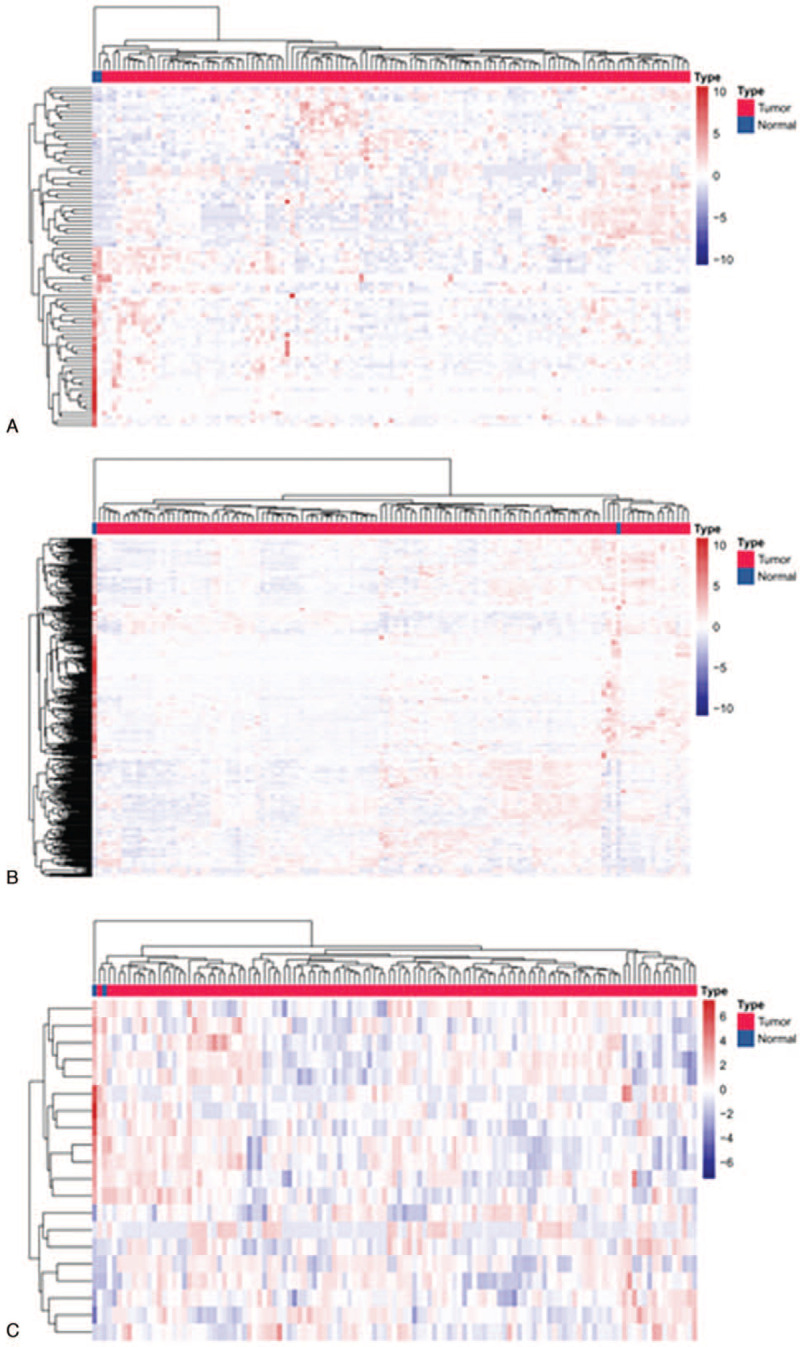
The heatmap represents hierarchical clustering for DEGs in TETs. (A) DElncRNAs. (B) DEmRNAs. (C) DEmiRNAs. DEGs = differentially expressed genes, DElncRNAs = differentially expressed lncRNAs, DEmRNAs = differentially expressed mRNA, DEmiRNAs = differentially expressed miRNA, TETs = thymic epithelial tumors.

Among the TETs datasets from UCSC Xena, a total of 6632 mRNAs, 2798 lncRNAs, and 316 miRNAs were obtained. We compared normal thymus with different pathologic types (A, AB, B, and TC) of TETs. In type A TETs, there were a total of 4265 DEmRNAs (2137 up- and 2138 down-regulated), 915 DElncRNAs (662 up- and 253 down-regulated), and 264 DEmiRNAs (120 up- and 144 down-regulated); For type B TETs, there were a total of 1996 DEmRNAs (887 up- and 1109 down-regulated), 313 DElncRNAs (122 up- and 191 down-regulated), and 113 DEmiRNAs (64 up- and 49 down-regulated) (Fig. 2A); In type AB TETs, there were a total of 2636 DEmRNAs (1197 up- and 1439 down-regulated), 1101 DElncRNAs (604 up- and 497 down-regulated), and 211 DEmiRNAs (106 up- and 105 down-regulated) (Fig. 2B); While in type TC TETs, there were 3003 DEmRNAs (1707 up- and 1296 down-regulated), 970 DElncRNAs (650 up- and 320 down-regulated), and 101 DEmiRNAs (42 up- and 39 down-regulated) (Log_2_FC > 1, FDR < 0.25) (Fig. 2C). The common DEGs of different pathologic types of TETs were shown in Fig. 3A–C and found the TETs samples could be easily recognized from the normal thymus tissues in the heatmap.

### 3.2 GO and KEGG pathway annotation of DEmRNAs

**Figure 4:**
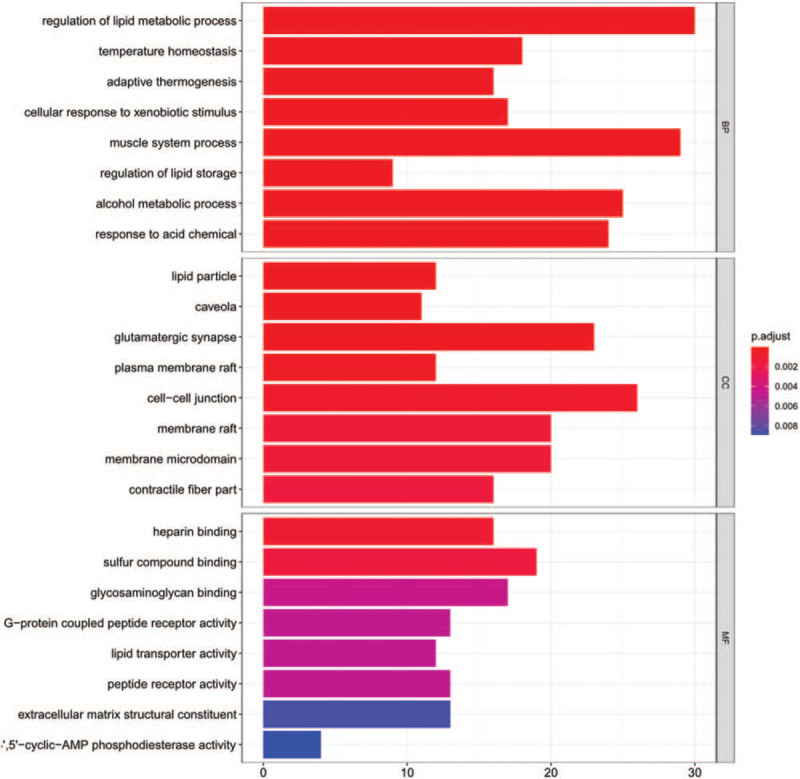
GO enrichment analysis for DEmRNAs in TETs, which divided into 3 categories (BP, CC, and MF, *P* < .05). BP = biological process, CC = cellular component, DEmRNAs = differentially expressed mRNA, GO = gene ontology, MF = molecular function, TETs = thymic epithelial tumors.

**Figure 5:**
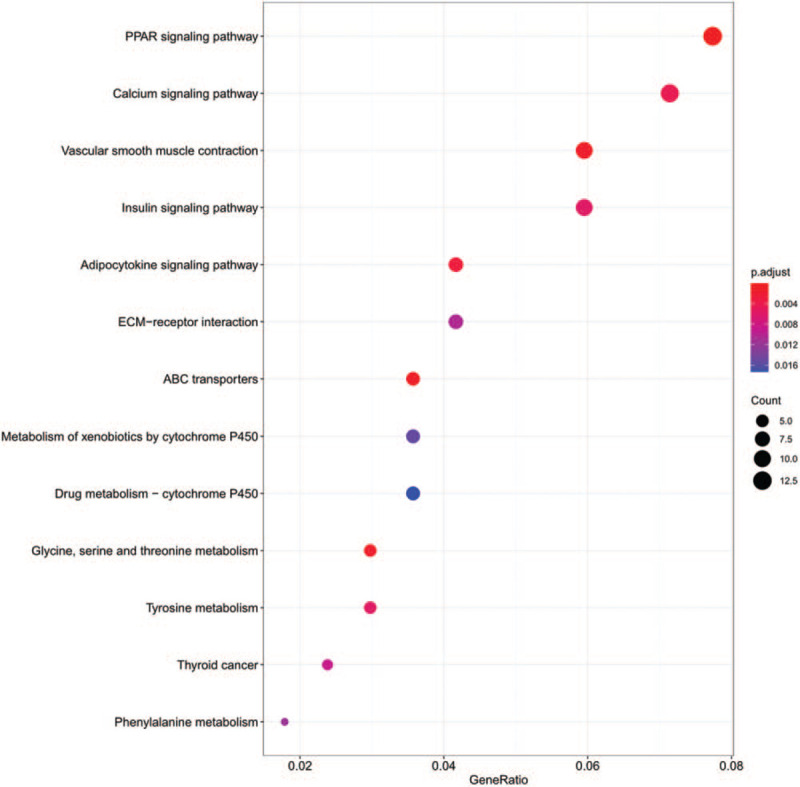
KEGG enrichment analysis for DEmRNAs. The top 13 KEGG pathway permutations according to the enriched number of DEGs (*P* < .05). DEGs = differentially expressed genes, DEmRNAs = differentially expressed mRNA, KEGG = Kyoto encyclopedia of genes and genomes.

A total of 463 abnormally expressed DEmRNAs were analyzed in this part. The enrich GO/KEGG function was used for GO and KEGG annotation. GO analysis could be divided into 3 functional groups: biological process (BP), cellular component (CC), and molecular function (MF) groups (Fig. 4). The most enriched GO biological process terms were “cell-cell junction,” “membrane raft,” “G-protein coupled peptide receptor activity,” and “extracellular matrix structural constituent” while the most significant KEGG pathways were “PPAR signaling pathway,” “Calcium signaling pathway,” “Vascular smooth muscle contraction,” “Insulin signaling pathway” (Fig. 5). Therefore, the functional enrichment results suggested that DEmRNA might be involved in the progression of TETs by regulating the above related biological processes and pathways.

### 3.3 Construction and analysis of ceRNA network in TETs

**Figure 6:**
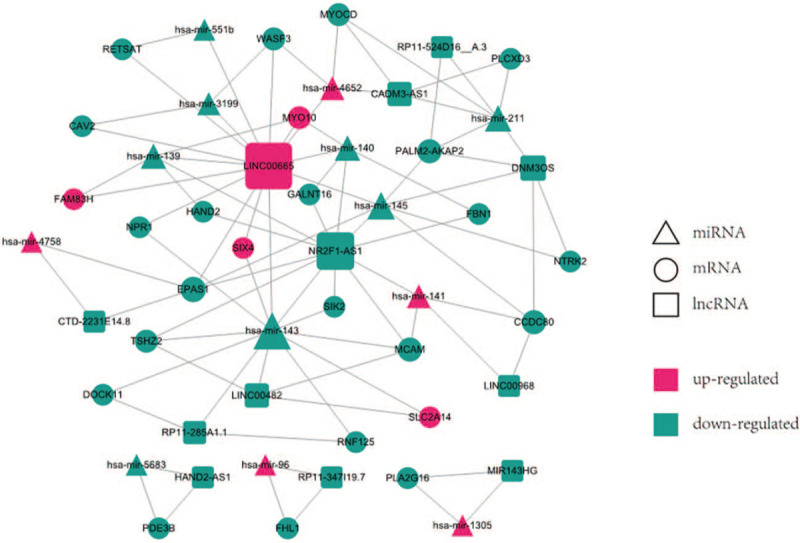
The lncRNA-miRNA-mRNA ceRNA network constructed from DEGs. Red nodes represent up-regulated genes and green nodes represent a down-regulated genes. Circles represent mRNA, rectangles represent lncRNA, and triangles represent miRNA. DEGs = differentially expressed genes.

To explore the potential relationship of DEGs, miRanda and starBase v2.0 database were used to predict the interaction of miRNAs with lncRNAs and mRNAs. According to the pairs of miRNA-mRNA and lncRNA-miRNA, an integrated lncRNA-miRNA-mRNA-based ceRNA network was built via Cytoscape software. The ceRNA network consisted of 12 lncRNA, 25 mRNAs, and 13 miRNAs, with a total of 50 nodes and 87 edges (Fig. 6). Based on the molecular level, lncRNA LINC00665, lncRNA NR2F1-AS1, lncRNA RP11-285A1.1 ranked the first, second, and fifth, respectively. mRNA MCAM, mRNA MYO10, mRNA WASF, and mRNA DOCK11 ranked fifth, sixth, sixth, and seventh, respectively.

### 3.4 GEO data verifies the expression of key ceRNA

**Figure 7:**
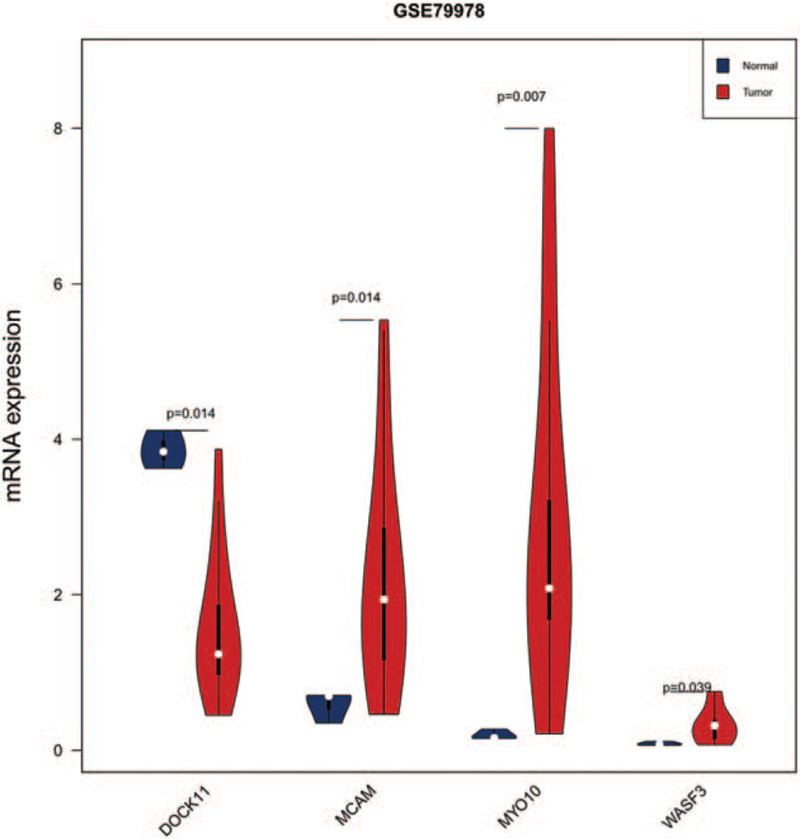
Expressions of DEmRNA (MCAM, MYO10, WASF3, and DOCK11) in an external cohort (*P* < .05). DEmRNAs = differentially expressed mRNA.

The high throughput sequencing profile GSE79978 of TETs from GEO database was utilized to validate the expression of key DEmRNAs in the ceRNA network. The mRNAs DOCK11, MCAM, MYO10, and WASF3 were proved to be consistent with previous studies. As shown in Figure 7 and Table 1, DOCK11 showed significantly lower levels in tumor datasets, while MCAM, MYO10, and WASF3 exhibited significantly higher levels in tumor datasets, compared with the normal group.

**Table 1:** DOCK11, MCAM, MYO10, and WASF3 expression in GSE79978 dataset.

	Thymic carcinoma vs normal	
Gene	log2FC	FDR
DOCK11	−1.27	0.014
MCAM	1.92	0.014
MYO10	3.95	0.007
WASF3	1.94	0.039

### 3.5 Survival analysis and establishment of the predictive model

**Figure 8:**
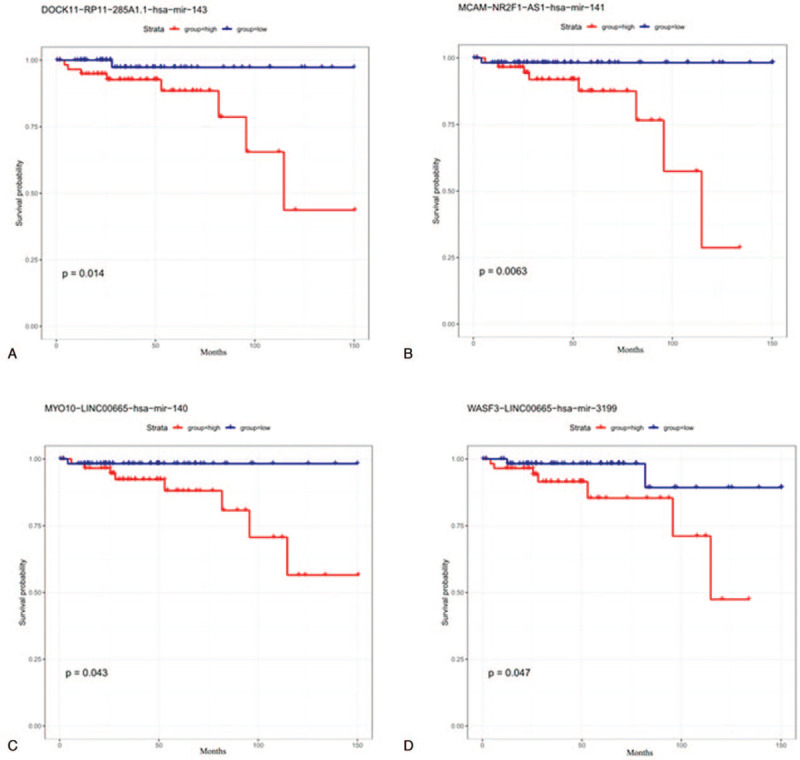
Kaplan–Meier survival curves for the 4 ceRNA modules associated with overall survival. (A) DOCK11-RP11-285A1.1-hsa-mir-143. (B) MCAM-NR2F1-AS1-hsa-mir-141. (C) MYO10-LINC00665-hsa-mir-140. (D) WASF3-LINC00665-hsa-mir-3199 (*P* < .05).

**Figure 9:**
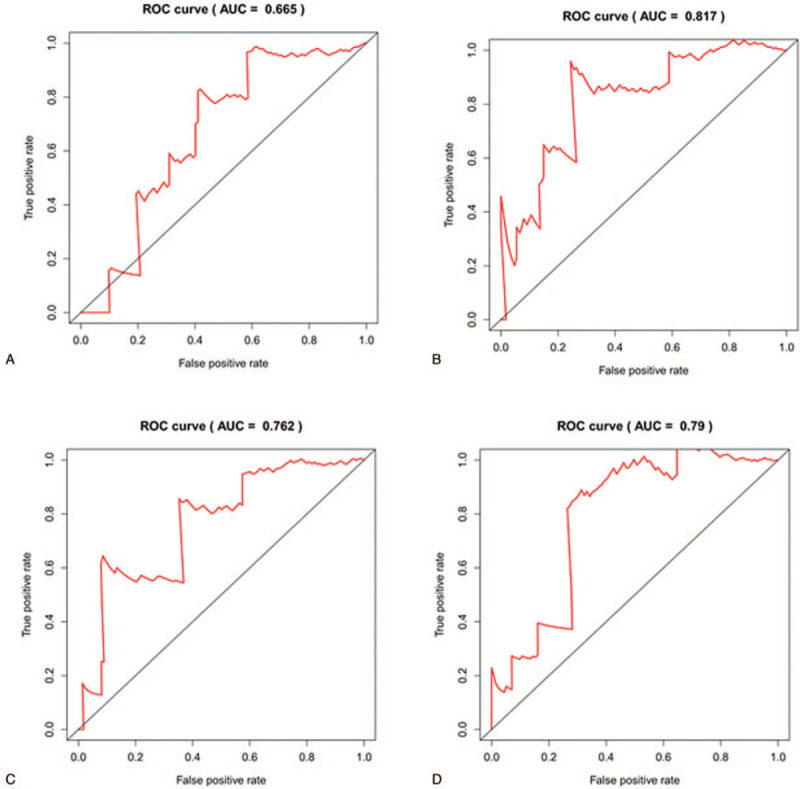
ROC curve for the 4 ceRNAs modules. (A) DOCK11-RP11-285A1.1-hsa-mir-143. (B) MCAM-NR2F1-AS1-hsa-mir-141. (C) MYO10-LINC00665-hsa-mir-140. (D) WASF3-LINC00665-hsa-mir-3199. (AUC > 0.65, *P* < .05). AUC = area under the curve, ROC = receiver operating characteristic.

To further study the relationship between ceRNA modules and prognosis of TETs, ceRNA modules and overall survival time were calculated. This significant prognostic-related lncRNA-miRNA-mRNA were further transferred to Cox regression analyses (*P* < .05). As shown in Figure 8 and Table 2, univariate Cox regression analysis indicated that DOCK11-RP11-285A1.1-hsa-mir-143, MCAM-NR2F1-AS1-hsa-mir-141, MYO10-LINC00665-hsa-mir-140, and WASF3-LINC00665-hsa-mir-3199 ceRNA modules were all associated with the overall survival of patients with TETs. The Kaplan–Meier survival curves suggested a positive correlation between DOCK11-RP11-285A1.1-hsa-mir-143 (Fig. 8A), MCAM-NR2F1-AS1-hsa-mir-141 (Fig. 8B), MYO10-LINC00665-hsa-mir-140 (Fig. 8C), WASF3-LINC00665-hsa-mir-3199 (Fig. 8D), and survival time. Our analysis showed the selected ceRNA modules could accurately predict survival probability (Fig. 9A to D). The AUC values of ROC curves of the 4 ceRNA modules were all greater than 0.65, indicating that the prognostic signature had a good prognostic effect.

**Table 2:** Gene fold change in 4 ceRNA pairs.

	Thymic carcinoma vs normal		typeA vs Normal		typeB vs Normal		typeAB vs Normal	
Gene	log2FC	FDR	log2FC	FDR	log2FC	FDR	log2FC	FDR
DOCK11	−2.58	4.11E−4	−2.25	1.44E−04	−1.4	1.23E−01	−1.48	2.2E−02
MCAM-	−2.75	3.62E−3	−1.61	1.38E−01	−2.94	4.43E−04	−2.7	1.55E−04
MYO10	2.44	2.26E−1	3.55	7.76E−06	2.49	1.28E−01	2.55	4.81E−03
WASF3	−2.16	2E−1	−1.98	1.84E−01	−2.75	7.65E−02	−2.27	7.76E−02
RP11-285A1.1	−1.895489	1.17E−1	−1.78	1.45E−02	−1.5	1.81E−1	−1.6	8E−02
NR2F1-AS1	−1.656851	2.36E−1	−2.28	1.45E−01	−3.19	9.01E−2	−2.33	1.56E−01
LINC00665	2.355539	1.66E−2	2.33	2.75E−08	1.62	6E−2	1.94	6.89E−08
hsa-mir-140	−2.178824	1.05E−2	−2.23	2.12E−04	−1.71	2.86E−03	−1.94	4.14E−06
hsa-mir-141	3.901336	7.81E−3	2.62	9.2E−04	2.37	4.67E−03	1.7	1.08E−01
hsa-mir-143	−2.953103	1.79E−4	−3.52	5.04E−05	−3.87	1.34E−05	−4.06	1.27E−08
hsa-mir-3199	−2.374443	7.48E−2	−2.57	2.93E−03	−2.65	4.92E−08	−2.39	2.63E−03

## 4 Discussion

TETs, as a kind of low incidence tumor, mainly originate from thymic epithelial cells. The pathological classification provides a powerful tool for grading patient's prognosis,^[17]^ in which TETs are further subdivided into thymomas (A, A/B, B1, B2, and B3 subtypes) and TCs. Thymomas are less aggressive and the 5-year survival rate for patients with thymoma is about 90%.^[18,19]^ While TCs is highly malignant and invasive and the 5-year survival rate of TCs is only 55%.^[20,21]^ Therefore, exploring the molecular pathological mechanism of TETs would help to provide a theoretical reference for the treatment of TETS in the future.

With the development of biotechnology, high-throughput sequencing technology and microarray had been applied to the research of TETs genome, such as epigenetic analysis, gene mutation detection, transcriptome analysis, and copy number variants.^[22–24]^ Recently, some researchers have revealed that some RNA molecules are widely involved in tumor initiation and progress. For example, circulating miR-21-5p and miR-148a-3p might act as a new biomarker in plasma to evaluate the efficacy and prognosis of TETs.^[25]^ Up-regulation of miR-145-5p could inhibit the proliferation and migration of TC1889 cells by inhibiting its target gene expressions.^[26]^

In this study, DEGs were screened by downloading the lncRNA, miRNA, and mRNA profiles of different pathologic types in TETs from the UCSC Xena database. A total of 2798 DElncRNAs, 316 DEmiRNAs, and 6632 DEmRNAs in the TETs group were distinguished. And 463 common DEmRNAs of different pathologic types in TETs were performed the functional enrichment analysis. The GO analysis showed that the DEGmRNAs were primarily involved in “cell-cell junction,” “membrane raft,” “G-protein coupled peptide receptor activity,” “extracellular matrix structural constituent..” KEGG analysis confirmed that “PPAR signaling pathway,” “Calcium signaling pathway,” “Vascular smooth muscle contraction,” “Insulin signaling pathway” might be involved in the development of tumors. In these results, PPAR signaling pathway had been proved that it might be a new molecular target for the treatment of TCs.^[27]^

An increasing number of studies have proved that ceRNAs are involved in the development of various tumors, and it could exert diverse biological functions in this process. ceRNAs regulatory network provides a new perspective to understand the relationship between miRNAs-mediated lncRNAs and mRNAs. Here, the ceRNA network of TETs was constructed, which may regulate the progression of TETs through a variety of mechanisms. In this ceRNA network, there were 50 nodes and 87 edges, including12 DElncRNAs, 13 DEmiRNAs, and 25 DEmRNAs. After verification with the external cohort, we found that DEmRNAs DOCK11, MCAM, MYO10, and WASF3 were consistent with the results from the training cohort. And finally, the 4 DEmRNA associated-ceRNA modules (DOCK11-RP11-285A1.1-hsa-mir-143, MCAM-NR2F1-AS1-hsa-mir-141, MYO10-LINC00665-hsa-mir-140, and WASF3-LINC00665-hsa-mir-3199) were selected for the follow-up study. In the ceRNAs network, we found that LINC00665 had the highest degree which may suggest its important role in TETs. lncRNA LINC00665 could bind with mRNAs MYO10 and WASF3 through miRNAs hsa-mir-140, hsa-mir-3199. This indicated that lncRNA LINC00665 might be the key molecule in the molecular regulatory mechanism of TETs. Studies had revealed that lncRNA LINC00665 was significantly upregulated in lung cancer and was an independent predictor of survival in lung cancer patients. And lncRNA LINC00665 could enhance the proliferation and invasion ability of lung adenocarcinoma cells via absorbing mir-98 and being involved in ERK signaling pathway.^[28]^ The lncRNA NR2F1-AS1 was the second degree in the ceRNA network, which could indirectly interact with the mRNAs FBN1, GALNT16, HAND2, and MCAM via hsa-mir-140, hsa-mir-139, and hsa-mir-141. Research had shown that NR2F1-AS1 upregulated FOXA1 through the adsorption of miR-483-3p, thus increasing the malignant degree of osteosarcoma and suggested it might be a potential therapeutic target for osteosarcoma.^[29]^

To further explore the biological function of the ceRNAs network, we used the Kaplan–Meier curve analysis to screen the DEGs related to the prognosis of TETs patients. Our analysis found that the above 4 ceRNAs modules, composed by 3 lncRNAs, 4 miRNAs, and 4 mRNAs., were significantly associated with the overall survival rate of TETs patients (*P* < .01). These 4 mRNAs MCAM, MYO10, WASF3, and DOCK11 were fifth, sixth, sixth, and seventh in the ceRNA network. As a new molecular marker, DOCK11 was reported to be highly expressed in TCs.^[30]^ MCAM, MYO10, and WASF3 were reported to be closely related to the regulation of the proliferation and metastasis of malignant tumors.^[31–33]^

However, our study still had some limitations. We just identified the ceRNA with mRNA as the target through the method of bioinformatic analysis without further experimental verification. Meanwhile, the 4 identified ceRNAs modules still need to be closely integrated with clinical practice to verify its reliability. To compensate for these shortcomings, we plan to collect TETs samples and relevant clinical information and conduct experiments to verify our results.

## 5 Conclusion

In this study, we analyzed the abnormal expression of key RNA molecules in different pathological types of TETs. Furthermore, the common DEmRNAs were used to conducted GO and KEGG enrichment analysis and revealed their potential biological function in TETs. A ceRNAs network was constructed based on the DEmRNA, DElncRNA, and DEmiRNA where 4 crucial DEmRNAs (DOCK11, MCAM, MYO10, and WASF) were screened and validated via an external cohort. The 4 ceRNAs modules associated with DOCK11, MCAM, MYO10, and WASF were closely related to the prognosis and survival of TETs which were verified via ROC analysis. In summary, these relevant ceRNA molecules not only provided the basis for the diagnosis and prognosis of TETs but also may be new therapeutic targets. Our study also further explored the molecular mechanism of RNA in TETs development, which would pave the way for future clinical practice.

## Acknowledgments

This work was supported by Fund of Key Laboratory of Myocardial Ischemia, Ministry of Education of China (KF201919).

## Author contributions

Guanghua Li designed experiments; Kegong Chen and Long Bai carried out experiments; Kegong Chen analyzed experimental results; Lin Ji and Libo Wu performed the research and acquired the data; Kegong Chen and Long Bai wrote the manuscript.

**Conceptualization:** Guanghua Li.

**Data curation:** Long Bai.

**Formal analysis:** Long Bai.

**Funding acquisition:** Kegong Chen.

**Investigation:** Lin Ji.

**Methodology:** Lin Ji.

**Project administration:** Lin Ji.

**Resources:** Lin Ji.

**Software:** Libo Wu.

**Supervision:** Guanghua Li.

**Validation:** Libo Wu.

**Visualization:** Libo Wu.

**Writing – original draft:** Kegong Chen.

**Writing – review & editing:** Kegong Chen.
